# Global health education produces healers

**Published:** 2016-12-05

**Authors:** Laurel Laakso

**Affiliations:** 1 Department of Community and Family Medicine, University of Toronto

She sent us an email before the session asking us to stereotype Indigenous people.

This email came to us a pre-session exercise. It required us to state three negative and three positive attributes that we immediately associated with Indigenous people. Then at the beginning of the session, she asked each of us — no hiding possible! — to share what we had written in the exercise.

The collective discomfort was palpable. Many of us residents sitting in the circle openly admitted that the things we had written were racist. No one had intended them to be, nor wanted them to be, and yet we were forced to acknowledge that our exposure to genuine First Nations, Inuit and Métis experience was limited. We had been forced to rely on stereotypes.

Our session presenter, a Canadian-trained indigenous physician, proceeded to lead our group of residents into the most open, most productive discourse on racism I had ever experienced. Racism is a determinant of health.^1^ Yet never once in my medical education had we ever talked about racism.

“I want this to be a safe place,” she told us. She quietly corrected misconceptions, and skillfully probed our assumptions. We talked about privilege. We talked about stereotyping patients in medicine under the pretense of facilitating a diagnosis. We talked about history. We talked about people who had died prematurely as a result of racism. We waded into waters that we would never have collectively explored without this level of guidance and safety.

This was only one of the many sessions I attended as a participant in the Global Health Education Initiative (GHEI). The GHEI is a two-year certificate program for medical residents and fellows led by Postgraduate Medical Education at the University of Toronto. It is not mandatory and prior to registering I had weighed the value, given the not insignificant investment of both time and money.

Yet the GHEI turned out to be a lifesaver. In the often-inhumane world of residency training — the hours, the stress, the inevitable lack of self-care — the GHEI provided a safe haven, a space where we could once again “be human.” We were each invited, session after session, to connect again to what it means to care for *people* — not just patients. To care for people who are members of a very large world. To care for people whose health is often the by-product of history, of systems, of attitudes.

When people talk about global health in medical education, both at the undergraduate and postgraduate level, I am stuck by how often it is portrayed as optional. It’s the icing on the cake, so to speak, a nice touch, but not what really matters. Yes, learning the science of medicine is first and foremost integral. But ultimately we are not scientists. We are doctors, and we care for people. Global health allows us to connect with this purpose, to reflect and understand the impact of who we are and what we do. It opens our eyes to see where we stand in a constantly changing and fluid world, and yet also reveals to us the intricate historical and social context in which we exist at any given time. It reawakens our humanity.

I recall that session on racism frequently. Beforehand, I had expected it to be a difficult, discouraging session, overshadowed by harsh, painful realities. Although the situations and circumstances we discussed were indeed harsh and painful, to my surprise the session itself was not. The discourse was hopeful, positive. Although real, it was also uplifting. Part of this was the role modeling of the session leader herself. I realize now what made the session so memorable; the session itself was *healing*.

Our fundamental identity as doctors, individually and collectively, is that of healer. That role applies not only to each unique patient but also to society as a whole.

Global health education enables us to experience, understand, and fulfill that role. It is integral to producing healers.
